# A restaurant-based intervention to promote sales of healthy children’s menu items: the Kids’ Choice Restaurant Program cluster randomized trial

**DOI:** 10.1186/s12889-016-2892-5

**Published:** 2016-03-10

**Authors:** Guadalupe X. Ayala, Iana A. Castro, Julie L. Pickrel, Christine B. Williams, Shih-Fan Lin, Hala Madanat, Hee-Jin Jun, Michelle Zive

**Affiliations:** San Diego State University, 5500 Campanile Drive, San Diego, CA 92182 USA; Institute for Behavioral and Community Health, 9245 Sky Park Court, Suite 221, San Diego, CA 92123 USA; Department of Pediatrics, Center for Community Health, University of California at San Diego, 9500 Gilman Drive, #0927, La Jolla, CA 92093 USA

**Keywords:** Restaurant, Children, Healthy menu, Employee training

## Abstract

**Background:**

Away-from-home eating is an important dietary behavior with implications on diet quality. Thus, it is an important behavior to target to prevent and control childhood obesity and other chronic health conditions. Numerous studies have been conducted to improve children’s dietary intake at home, in early care and education, and in schools; however, few studies have sought to modify the restaurant food environment for children. This study adds to this body of research by describing the development and launch of an innovative intervention to promote sales of healthy children’s menu items in independent restaurants in Southern California, United States.

**Methods:**

This is a cluster randomized trial with eight pair-matched restaurants in San Diego, California. Restaurants were randomized to a menu-only versus menu-plus intervention condition. The menu-only intervention condition involves manager/owner collaboration on the addition of pre-determined healthy children’s menu items and kitchen manager/owner collaboration to prepare and plate these items and train kitchen staff. The menu-plus intervention condition involves more extensive manager/owner collaboration and kitchen staff training to select, prepare, and plate new healthy children’s menu items, and a healthy children’s menu campaign that includes marketing materials and server training to promote the items. The primary outcome is sales of healthy children’s menu items over an 18-week period. In addition, dining parties consisting of adults with children under 18 years of age are being observed unobtrusively while ordering and then interviewed throughout the 18-week study period to determine the impact of the intervention on ordering behaviors. Manager/owner interviews and restaurant audits provide additional evidence of impact on customers, employees, and the restaurant environment. Our process evaluation assesses dose delivered, dose received, and intervention fidelity.

**Discussion:**

Successful recruitment of the restaurants has been completed, providing evidence that the restaurant industry is open to working on the public health challenge of childhood obesity. Determining whether a restaurant intervention can promote sales of healthy children’s menu items will provide evidence for how to create environments that support the healthy choices needed to prevent and control obesity. Despite these strengths, collection of sales data that will allow comprehensive analysis of intervention effects remains a challenge.

**Trial registration:**

NCT02511938

## Background

Trends in eating food-away-from-home (FAFH) suggest children are eating more FAFH than ever before [1], with FAFH spending reaching a level nearly equal to food-for-home spending in 2014 [2]. Children’s daily caloric intake from FAFH increased from 23 to 34 % between 1977 and 2006, reflecting an increase from 447 to 702 calories (cal) from FAFH [1]. This is concerning given that multiple studies have found the nutritional quality of most restaurants’ children’s menu items do not meet nutritional standards [3–5]. As such, FAFH consumption has negative implications on children’s diet quality [6], with studies finding FAFH is associated with more consumption of energy, fat, added sugars, and sugar-sweetened beverages, and less consumption of milk, fiber, and fruits and vegetables (FVs) [7, 8]. In addition, at least weekly or more frequent consumption of FAFH is associated with obesity risk for United States (US) residents [9, 10] and elsewhere [11, 12], including in the present study community [13].

Responding to public health concerns, the National Restaurant Association started the Kids LiveWell program [14], an effort encouraging 42,000 predominantly chain restaurants to offer at least one full children’s meal (including side dish and drink) and one children’s menu item that meet nutritional standards. Despite this national effort, lacking are rigorous studies identifying the most effective strategies for promoting the sale of healthy children’s menu items in independent restaurants, which represent 66 % of all restaurants in the US [15]. Furthermore, restaurants offering ethnic foods are more likely to be independent [15], thus attracting segments of the population with the greatest health disparities (e.g., immigrants) [16, 17].

### Restaurant interventions

Restaurant interventions targeting children’s menu items are an innovative and potentially significant approach for improving the food environment [18], and consumption among dining parties [19]. For example, menu-labeling interventions have observed trends in decreasing calories purchased [20–22]. Other strategies also showed promise. In 13 outlets of one large chain restaurant, a menu modification intervention (i.e., 100 % of children’s meals included a healthy side dish and a healthy beverage) resulted in significantly fewer calories ordered by children who accepted the healthier default side and increased revenue for the restaurant chain [23]. More generally, a 2015 review of 27 restaurant interventions concluded that the only intervention strategies with some evidence are those that involve increasing the availability of healthy items on the menu and promoting these items through various modes or channels (e.g., point-of-purchase signage) [19]. However, most of the identified studies (20 of 27) did not meet criteria for inclusion in the review given weak study designs and lack of outcome data.

### Present study

Lacking are rigorously-designed studies evaluating methods for promoting healthy children’s menu items in independent restaurants. Most previous efforts have focused on chain restaurants [14]. The Kids’ Choice Restaurant Program (KCRP) is testing a multi-component intervention to promote the sales of healthy children’s menu items in independent restaurants in San Diego, CA. This paper describes the development of KCRP and presents baseline characteristics of the restaurants and managers/owners.

## Methods/Design

### Overview and study design

Consistent with CONSORT guidelines [24], KCRP is a cluster randomized trial with eight pair-matched independent restaurants randomized to a menu-only versus a menu-plus intervention condition. Outcome and impact evaluation activities are designed to determine whether the interventions result in sales of healthy children’s menu items and changes in ordering behavior among dining parties with children. Weekly sales data are collected from restaurant managers/owners for 18 weeks, including 4 weeks pre-intervention (baseline). Unobtrusive observations are attempted with approximately five dining parties with children every other week for 18 weeks in each restaurant to capture ordering behavior. Post-order and post-meal interviews with these same dining parties, as well as some unobserved dining parties, capture reported ordering and consumption behaviors. An extensive process evaluation protocol, including ongoing documentation by KCRP intervention staff, restaurant audits and manager/owner interviews, assesses key dimensions of implementation [25], including dose delivered, dose received, and intervention fidelity. Employees are recruited to participate in the restaurant training; however, the only evaluation activity they are involved in is assessment of the training. This study was conducted according to the guidelines laid down in the Declaration of Helsinki and all procedures involving human participants were approved by the Institutional Review Board of San Diego State University.

### Study foundation

The KCRP protocols are informed by formative research with restaurants in San Diego, CA, a pilot study in Imperial County, CA [26], the *Aventuras para Niños* restaurant intervention [27], input from members of the restaurant industry (see acknowledgements), as well as previous intervention research targeting changes to the food store environment [28–31]. The formative research study identified gaps in customer service, an issue important to restaurant managers/owners [32]. It confirmed the need to design materials that appeal to both parents and children given differences observed in ordering behaviors among older children. The pilot study confirmed the need to be prepared to take action with the restaurants immediately upon study enrollment versus waiting for the researchers to prepare their protocols, obtain institutional review board approval, etc. In other words, once they agreed, restaurant managers/owners were ready to begin study activities [26]. The *Aventuras para Niños* intervention identified the need to keep the intervention simple given high demands on manager/owner and restaurant staff time and effort [33]. Restaurant industry representatives informed the content and design of recruitment protocols and intervention strategies relevant to the restaurant managers/owners and customers involved in the present study. Our interventions are based on the integration of behavioral, organizational, and structural change theories [34–36], all of which fit under the Socio-Ecological Framework (SEF), which considers multiple sources of influence on health behaviors and health outcomes [37, 38]. SEF acknowledges the importance of organizational and structural models to inform intervention efforts to improve access to, and availability and promotion of, healthy foods and beverages [8, 39].

### Restaurant selection, setting, and intervention condition assignment

Restaurant eligibility included being a full-service restaurant (i.e., not quick service or buffet only); having a minimum of 20 tables; offering American or Latino food (i.e., the latter including but not limited to foods/beverages from Mexico, Puerto Rico, Cuba, Central and South America); and having sufficiently detailed receipts for coding purposes and/or the ability to provide detailed sales data. The first two criteria ensured that ordering would occur from among a large enough sample. Type of food was limited to minimize sources of variance such as sharing of meals, commonly observed in Italian and Chinese restaurants. Restaurant manager/owner eligibility criteria included being at least 21 years of age; having worked at least 20 hours per week for the participating restaurant for a minimum of 4 months; planning to continue working as restaurant manager/owner for the study duration; having decision-making authority in the restaurant; willingness to provide sales data to researchers; and not being employed at another participating restaurant.

**Fig. 1:**
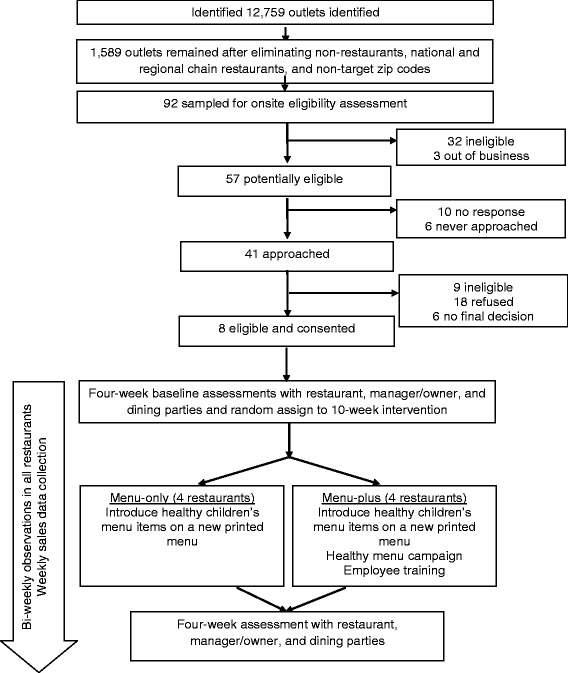
Study design

Identification of permitted restaurants was conducted using February 2014 San Diego County Department of Environmental Health food permits, which yielded a list of 12,759 food outlets. After removing 5112 food establishments that were clearly not restaurants (e.g., lodging, grocery stores, convenience stores), and 1841 known national and regional chain restaurants (e.g., McDonalds), the list was reduced to 5806 outlets. This list was further reduced to a combined list of 1589 restaurants by targeting zip codes within a 5-mile radius of study offices and zip codes between 5 and 10 miles of study offices with a Latino population of at least 29 % using 2010 US Census data [40]. From this list, 92 American and Latino restaurants were identified as potentially eligible (see Fig. 1).

Initial restaurant recruitment strategies involved a phone call or drop-in visit to schedule an in-person meeting with the restaurant manager/owner. If restaurant employees informed the KCRP recruiter that the restaurant manager/owner should be contacted first via email, an email with the introductory letter was sent. During the recruitment meeting with the manager/owner, the KCRP recruiter reviewed study requirements and gave the manager/owner a study fact sheet with testimonials from managers/owners who participated in the pilot study and answers to frequently-asked questions. In addition to learning more about the study, the manager/owner was told his/her restaurant would receive $300 for participating, the manager/owner would receive $20 for each of two interviews completed, and wait staff and customers would receive $10 per shift and $5, respectively, the former for allowing KCRP evaluation staff to shadow the wait staff and the latter for completing the interviews. If the manager/owner was interested, the KCRP recruiter completed eligibility screeners for both the restaurant and the manager/owner. If eligibility was met and the manager/owner agreed, the two parties signed a letter of agreement and confidential disclosure agreement. The manager/owner also signed an informed consent form for the interviews. Restaurants were pair-matched on availability of a children’s menu (yes or no) and with one exception, on size (small-to-medium or medium-to-large based on number of tables). The exception involved one set of pair-matched restaurants both without a child menu but of different sizes. Once a pair was identified, the project biostatistician randomly assigned each restaurant to either the menu-only or menu-plus intervention condition and the intervention planning and development phase for the pair commenced. KCRP evaluation staff blinded to intervention condition assignment completed baseline measures.

### Intervention conditions

In both the menu-only and menu-plus intervention conditions, the children’s menu intervention is implemented over a 10-week period. This occurs after an initial start-up period of about 6 weeks during which KCRP intervention staff work with the restaurant manager/owner to decide upon the various intervention components. In both intervention conditions, restaurants are asked to consider children’s menu items that fit their current inventory to minimize the number of new ingredients needed. Restaurants with an existing children’s menu are encouraged but not required to remove existing menu items from their offerings.

#### Menu-only intervention condition

Restaurants randomly assigned to the menu-only intervention condition are provided a standard set of healthy children’s menu items to include in their offerings on a new color-printed and laminated children’s menu provided by the study (see Table 1). All meals meet nutritional standards that reduce calories, fat and sugar compared with traditional children’s menu items. To do this, the following strategies are used: (a) decreasing portion sizes of existing and proposed menu items, an important though challenging target for change [41]; (b) adding fruit and vegetable side dishes to the menu, sometimes with serving sauces, which is one method for overcoming food neophobias that children may have to new foods and beverages [42]; and (c) establishing healthy side dishes as the default, which may reduce calorie intake by up to 170 cal [23]. Previous research has shown small changes in default foods can improve the nutritional value of foods promoted to children, especially if an indulgence is maintained (e.g., smaller sized french-fry portion plus apple slices) [43]. Children have neutral or positive attitudes toward these changes [4]. Thus, consistent with the concept of behavioral economics to automatize the selection of healthier versus less healthy items [23], and because of the importance of having choices and being flexible [32], our children’s meals include a healthy side dish as a default and sometimes a healthy beverage (i.e., 1 % or skim milk; 100 % orange or apple juice). Importantly, only healthy beverages are included on the new children’s menus; however, per owner/manager preferences, substituting a previously available side dish or beverage is possible. To finalize the menus, decisions are made about their pricing and plating. Given parent and child preferences for “all-in-one” pricing (e.g., main dish, side and beverage) and pricing that is similar to unhealthy menu items [42], managers/owners are encouraged to set an “all-in-one” price and to be consistent with the pricing of other children’s menu items. How foods are presented (e.g., food appearance and portion size) [5, 44, 45] influences choice, thus it is suggested to use smaller plates to retain a pleasing ratio of food to “white-space” [46].

**Table 1:** Intervention components and associated process evaluation measures for menu-only and menu-plus conditions

	Planned dose	Process evaluation
Menu-only		
New healthy children’s menu	Five standard healthy children’s meals that meet the following criteria in combination with side dish and beverage offerings: no more than 600 cal; no more than 50 % calories from fat; no fried foods; child-appropriate portion sizes; fruit or vegetable side dishes; healthy beverages (1 % or nonfat/skim milk and/or 100 % fruit juice [e.g., orange or apple])	# of main dishes implemented # of side dishes implemented # of healthy beverages implemented Mean calories per meal combination available $ spent on kitchen supplies to prepare new items Retention of existing children’s menu items Mean amount of time spent on kitchen manager meeting
	Kitchen manager meeting to prepare new recipes	
Menu plus		
New healthy children’s menu	Same as menu-only with more flexibility in terms of choices offered	Same as menu-only
Marketing campaign	One banner	% (n) of banners installed and retained at each weekly visit; placement of banners % (n) of table tents installed and retained at each weekly visit # of customers observed looking at KCRP materials Impact of design elements on sales
	Table tents (quantity based on number of tables)	
Trainings	All servers, bus-persons and hosts/hostesses invited to receive 15-min introductory training	Median (range) and % of servers, bus-persons and hosts/hostesses who received 15-min introductory training % of those attending introductory training who also received advanced training % of observations in which server was observed prompting use of KCRP materials # of pocket guides distributed at trainings % of kitchen employees who received kitchen staff training % of training content that was completely covered for all trainings
	At least 50 % of servers, bus-persons and hosts/hostesses who attended introductory training invited to attend 15-min advanced training	
	All kitchen staff invited to receive 15-min kitchen staff training	

Following selection of menu items, a KCRP intervention staff member meets with the kitchen manager who is responsible for training the kitchen staff to adopt the new menu items for at least 10 weeks. Kitchen managers/chefs work with a KCRP intervention staff member on the preparation and plating of the new children’s menu items during one meeting lasting between 45 and 90 minutes (min). A key focus of this meeting is on food portion sizing for children. Kitchen managers/chefs then train the kitchen staff on the preparation of the new items.

**Fig. 2:**
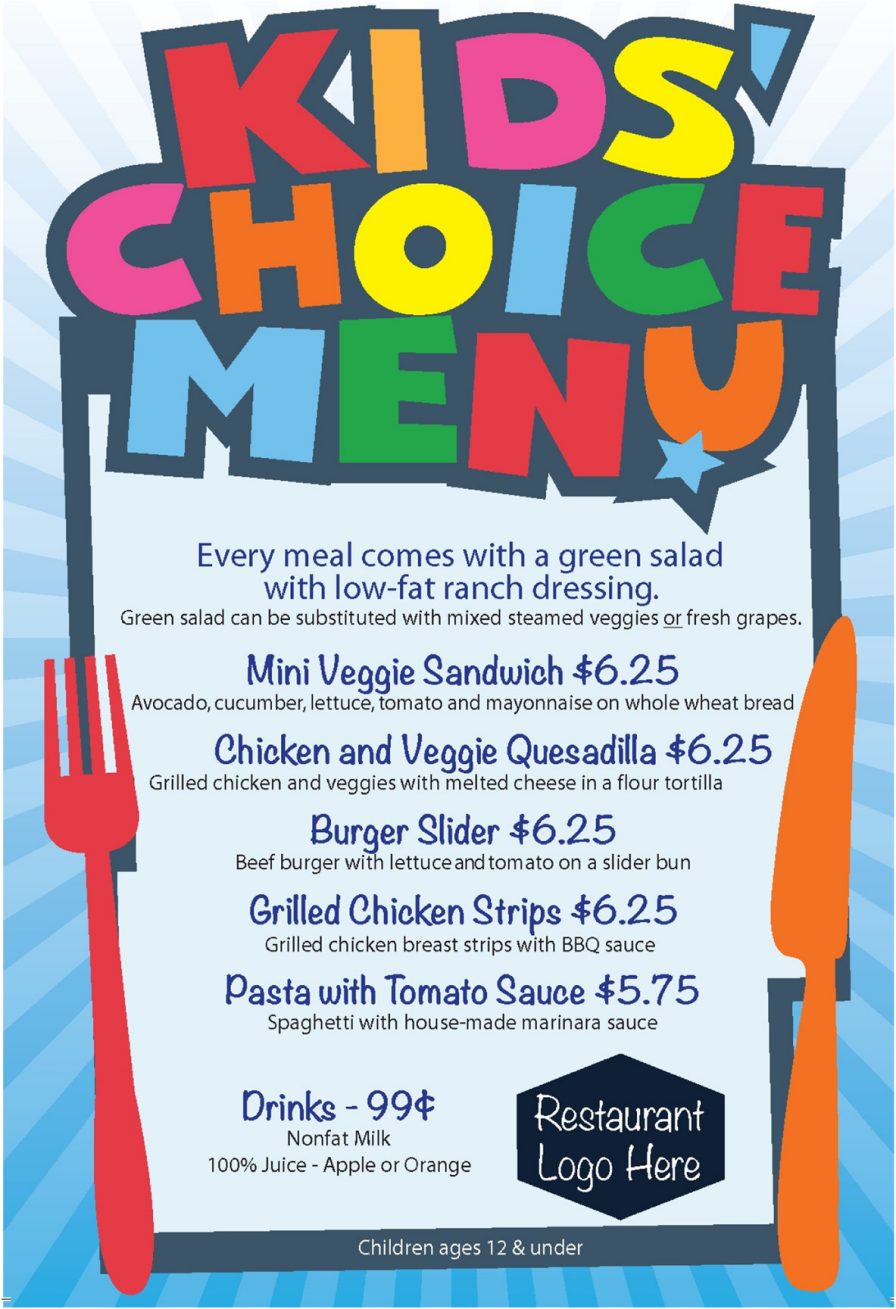
New healthy children’s menu for menu-only condition

The healthy children’s menu in the menu-only intervention condition includes five main dishes, at least three side dishes and two beverages (see Fig. 2). The nutritional quality of each meal combination is evaluated against current nutritional standards [14, 47, 48]. Meals, including a healthy side dish and a healthy beverage, range from 142 to 601 cal. Main dish items are between 37 and 366 cal. Since restaurants are not required to remove existing children’s menu items or modify any aspect of adult menu items, children could substitute existing side dishes and beverages when ordering a new children’s meal. New children’s menus are printed with the restaurant and project logos and include the price and what the price includes (e.g., with or without a beverage). However, unlike the menu-plus intervention condition as described below, meal names are presented in a randomly ordered list. Printed menus are distributed to dining parties consistent with restaurant practices.

#### Menu-plus intervention condition

**Fig. 3:**
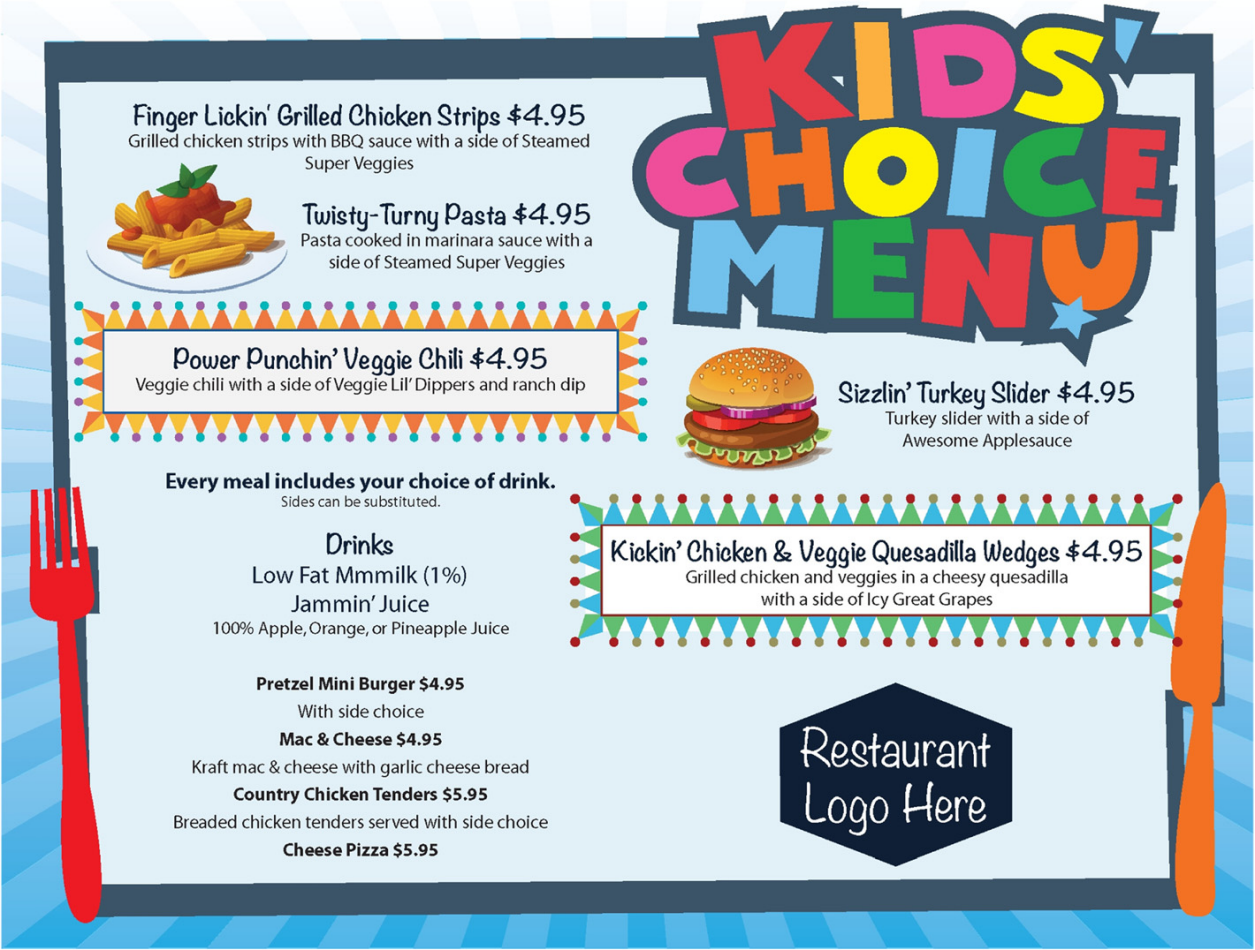
New healthy children’s menu for menu-plus condition

Similar to a previous study [49], restaurants randomized to the menu-plus intervention condition are engaged in a more collaborative process of: (a) identifying healthy children’s menu items that may fit better with their existing adult and children’s menus (if available); (b) implementing a healthy children’s menu campaign that includes a printed children’s menu with copy and design elements that influence ordering behavior [50], a banner and table tents; and (c) kitchen staff and server training to prepare and promote the new healthy children’s menu items (see Table 1). Regarding the menu items, to maximize the sales of new children’s menu items, decisions about what items to include, their recipes, prices, etc., are made over three to four meetings with the restaurant manager/owner. It is important to get manager/owner buy-in to maximize implementation fidelity [51]. Regarding the campaign, the menus use copy and design elements that have been shown to capture consumers’ attention, including fun names and descriptions of the new menu items, placement of new menu items in specific locations on the menu, and boxing menu items and including pictures to draw attention to them (see Fig. 3) [50]. In short, the menu-plus intervention condition involves social and structural changes in the restaurants, with activities directed at the managers/owners, employees, and customers. Research-supported intervention activities geared toward development of the menus and campaign occur over several weeks immediately prior to the 10-week customer-directed activities.

#### Healthy menu campaign

The goal is to change ordering behavior through a healthy menu campaign. Outside banners are used to draw dining parties with children into the restaurants. Table tents and banners are used to promote the menu items, similar to previous research [49]. The materials are designed to appeal to both children and adults given the increased autonomy children exhibit in restaurant ordering, compared with other food-related decisions [42].

#### Employee trainings

In addition to the kitchen manager meetings, in the menu-plus intervention condition, kitchen staff receive a 15-min training introducing the KCRP project and the new children’s menu items in their proper portion and plating arrangements. Servers, bus-persons and hosts/hostesses also receive one 15-min introductory training, and a subset of servers, bus-persons, and hosts/hostesses participate in a second 15-min training a few weeks later. Using a script and slide presentation, KCRP intervention staff train the employees on the following topics: customer service and suggestive selling, interacting with dining parties with children, and promoting the new children’s menu items. Server training was identified as an important strategy for creating a stress-free environment, a factor identified as important to parents with young children when selecting a restaurant [42, 52]. These trainings occur during the development phase and prior to implementation of the new children’s menu and the healthy menu campaign. Optimal delivery of the trainings occurs in small groups; however, they can be delivered one-on-one. Trainings include opportunities for role playing and skill practice consistent with behavioral modification principles [53]. At the end of the introductory training session, employees are given a pocket guide to remind them of the key points of the training and provided strategies.

### Intervention implementation

The interventions are initiated once all baseline data are collected, menu items are selected, menus are finalized, and trainings are completed; for each pair-matched restaurant, interventions start on the same day. After the new children’s menus are introduced (along with the healthy menu campaign in the menu-plus intervention condition), a KCRP intervention staff member conducts on-site support visits with each restaurant to monitor implementation and help restaurant managers/owners address any issues that may arise with the new children’s menus. These visits occur three times over the 10-week intervention for menu-only restaurants and weekly for menu-plus restaurants. Support visits consist of informal meetings lasting approximately 15 min that are guided by a set of open-ended questions and allow for discussion of any outstanding issues that need to be addressed. A KCRP intervention staff member monitors implementation to maximize implementation fidelity, addressing concerns with the manager/owner either immediately or soon after they are identified.

### Evaluation

#### Overview

A mixed-methods approach is used to evaluate the intervention on sales of healthy children’s menu items over an 18-week period, including during the 4-week baseline period. Primary outcomes are gross sales of healthy children’s menu items and units sold. Observations and interviews with a convenience sample of dining parties with children below the age of 18 years will determine changes in ordering behavior. Manager/owner interviews conducted during the baseline period and during the final 4 weeks of the 18-week data collection period (herein referred to as post-intervention period), and restaurant audits at baseline, mid-point of intervention and post-intervention periods will provide evidence of intervention impact on the manager/owner and restaurant environment. Our process evaluation includes an additional manager/owner interview once intervention activities are completed (i.e., exit interview). KCRP intervention staff visit the restaurants to capture implementation, dose delivered, dose received, and fidelity, and manager/owner and customer satisfaction with the intervention (see Table 1).

#### Primary outcome evaluation

Sales in gross dollars and units sold are tracked for existing and new children’s menu items, as well as total gross food and beverage sales per week excluding alcohol. Weekly detailed sales data are collected for an 18-week period, starting during the 4-week baseline period. Managers/owners are given a sales data guide to help them prepare the data to maximize its utility for the study. In addition, reminder emails/phone calls are used to maximize getting the data in paper or electronic form. These data have the potential to yield information on calories sold in the form of new children’s menu items; however, funding limits prohibit us from evaluating the nutritional quality of existing children’s menu items.

#### Secondary outcome evaluation

Biweekly observations and interviews with dining parties occur in each restaurant over the 18-week period; 45 observations/interviews are planned for each restaurant. Dining parties are observed unobtrusively if they include at least one child who appears to be below the age of 18 and at least one adult. A trained KCRP evaluation staff member, acting as a server-in-training, captures the language of the interaction with the server, what is ordered for each child, and who places the order. All food and beverage orders for/by children are captured; however, if a child is sharing a meal with another child, the order is captured only once for/by the child who ordered. At the conclusion of the observation, the KCRP evaluation staff member also notes whether the server interacts with children directly, recommends food and/or beverage products off the children’s menu(s), or prompts use of any KCRP materials, and if the customers ask the server about KCRP or are observed looking at any of the KCRP materials.

After the orders are placed, a second trained KCRP evaluation staff member visits each table to conduct an immediate interview about ordering behavior and a second interview post-meal about consumption behavior. The former captures information on composition of the party, how frequently the dining party visits the restaurant, whether they are celebrating a special occasion, what was ordered and who placed the order for each child, and what influenced the ordering behavior (e.g., use of menus, other marketing materials, etc.), including whether the server suggested any menu items. Information on sharing of food and beverages is also captured. The post-meal interview captures information about how much was consumed, child satisfaction with the food, and adult satisfaction with the children’s menu items. To obtain consent for interviews, observed parties are handed an informational flyer and an informed consent/recruitment script is read by KCRP evaluation staff who then asks if they would be willing to participate. As soon as an adult member of the dining party agrees to participate, KCRP evaluation staff screens the dining party for eligibility: at least one adult 18 years of age or older and one child under the age of 18. If the dining party is eligible and agrees to participate, KCRP evaluation staff collects the post-order and then post-meal interview, collecting information for each child in the party. If the dining party refuses at any point during recruitment, the refusal is noted and no additional data are collected. If the dining party is ineligible, the party members are thanked for their time and ineligibility criteria are noted.

Managers/owners are interviewed three times over the course of their involvement: during the 4-week baseline period, within three weeks after the 10-week menu implementation period (exit interview), and during the 4-week post-intervention period. The baseline and post-intervention interviews are similar to assess changes in managers’/owners’ perceptions about the restaurant’s ability to promote healthy children’s menu items (six-item scale, 5-point Likert-type response options) and perceived barriers to implementation (nine-item scale, same response options). The baseline interview also includes an eight-item scale assessing the restaurant’s readiness to implement KCRP (same response options), as well as questions about the manager/owner such as his/her age, gender, race/ethnicity, years of education, employment history with the restaurant, and level of decision-making control. Several questions ask about the restaurant, such as the number of full- and part-time employees by job title. The post-intervention interview includes additional questions about managers’/owners’ perceptions of the various intervention components, including whether the KCRP intervention improved aspects of the restaurant, such as profitability and customer service (seven-item scale, same response options). Changes in the restaurant environment, including availability and promotion of foods and beverages, are captured at baseline, mid-intervention, and post-intervention with a restaurant audit tool adapted from the Nutrition Environment Measurement Survey for Restaurants (NEMS-R) [54] and the Children’s Menu Assessment (CMA) [55].

#### Process evaluation

KCRP intervention staff members document resources needed to implement the new healthy children’s menu items. This includes information on what new foods and beverages the restaurant needs to purchase, and what new cooking and serving equipment are needed for each children’s menu item. KCRP staff monitor intervention implementation during check-in visits (three times during the 10-week intervention period in the menu-only restaurants and weekly in the menu-plus restaurants). In addition to assessing the overall restaurant environment (e.g., whether a special event is occurring, number of dining parties with children), the intervention staff member documents the quantity of KCRP children’s menus available, including any in use by dining parties with children, as well as banners and table tents in the menu-plus intervention condition. The intervention staff member also notes whether other children’s menu promotional activities are occurring (e.g., a special promotion) and/or whether any other healthy eating promotions are occurring.

Implementation of the trainings is assessed in three ways: **reach,** or the number of employees trained by gender and job title based on training sign-in sheets maintained by the intervention staff member; **dose delivered;** and **satisfaction.** Dose delivered is assessed both objectively in terms of the number of minutes of training time delivered to each restaurant by type of training, as well as subjectively, including trainer self-assessment of each training on: (a) the extent of content covered using an eight- to 13-item scale consistent with training content, (b) level of trainee engagement in each aspect of the training on a seven- to 12-item scale, and (c) conduciveness of the environment for the training using a four- to five-item scale. In terms of satisfaction, trainees are asked to complete a four-to-five item participant satisfaction survey at the end of training. In addition, satisfaction with all aspects of the intervention is assessed during the manager/owner exit interview (qualitatively) and post-intervention interview (quantitatively). Dining parties are asked about their satisfaction with the new main dishes, side dishes and beverages, as well as the taste of the food during the post-meal interview.

### Planned analyses

All analyses will be carried out according to the intention-to-treat rule consistent with standard practice in most trials. The primary aim of this study is to assess the impact of the intervention on sales of healthy children’s menu items. The primary dependent variables (gross sales in dollars and units) are continuous measures that are normally distributed or may be transformed to approximate normality by natural log or the Box-Cox transformation. Thus, normal-based models will be emphasized, with care taken to assess this assumption and, if necessary, apply appropriate transformations. Based on bivariate analyses (independent sample *t*-test and chi-square test), significant differences between the two intervention conditions will be included in the subsequent multivariate mixed model analysis for covariance adjustment.

For the primary aim of assessing the effectiveness of the interventions on sales of healthy children’s menu items, we will use mixed effects models. The primary predictor, intervention condition assignment, along with sales over time between the two intervention conditions will be placed in the fixed effect part of the models. These covariates will be adjusted for their influence on the intervention effect. The restaurant ID will be placed in the random effect part of the model to adjust for the clustering effects. The proposed multilevel models will be fitted using SAS PROC MIXED or PROC GLIMMIX when examining non-normal variables such as a dichotomous outcome.

## Discussion

From among the 92 potentially eligible restaurants, 32 were ineligible prior to approaching the manager/owner for not being a full-service restaurant, having too few tables, or not having the capability to provide sales data. From the remaining 57 restaurants, 10 never responded to any recruitment attempts and six were never approached due to completion of recruitment. Forty-one restaurants were approached in-person for participation; nine were ineligible, 18 refused to participate, six never provided a final decision, and eight agreed (see Fig. 1). Among the reasons given for participating, managers/owners noted that they were interested in providing healthier items for children. Among reasons for refusing, managers/owners said they were too busy or not interested, their restaurants were undergoing major changes during the upcoming months, or the timing of the study necessitated more extensive management involvement than they could provide during the peak tourist season. However, other managers/owners reported they were not interested in “telling customers how to eat” or did not want to change their existing menus.

Restaurants are pair-matched at recruitment to ensure equivalency across characteristics randomized to intervention conditions (e.g., presence of a children’s menu). Thus, once a restaurant is recruited, a second restaurant from the list is recruited with similar characteristics. One restaurant is randomly assigned to the menu-only intervention condition and the other to the menu-plus intervention condition. Baseline assessments take place during the 4 weeks prior to the introduction of the new children’s menus and are conducted by KCRP evaluation staff blind to treatment condition.

### Baseline characteristics

Depicted in Table 2 are manager/owner and restaurant characteristics at baseline. Restaurant managers/owners are mostly male (88 %) and all had some college education. Average years managing the restaurant are 12 (SD = 8) and the managers/owners reported working, on average, over nine hours a day (SD = 2). As desired, the eight restaurants are all independent, with two of the owners having a partnership in two other restaurants. One matched pair did not have a children’s menu at baseline; the remaining six restaurants did. The average number of full-time employees is 13 (SD = 7), average number of tables is 33 (SD = 7), and 75 % typically have 60 or more dining parties on a busy night. Average weekly sales are $21,500 (range = $8000 to $60,000).

**Table 2:** Manager/owner and restaurant characteristics (*N* = 8)

	%, Mean, or median	N, SD, or range
Manager/Owner characteristics		
Mean age in years	50.6	12.2
Male	87.5 %	7.0
White/Caucasian	71.4 %	5.0
Some college	100.0 %	8.0
Mean years managing this restaurant	12.2	8.0
Mean hours worked in a typical day	9.4	2.3
Restaurant characteristics		
Mean years in operation	17.8	15.2
Mean number of full-time employees	12.9	6.6
Mean number of part-time employees	11.1	11.5
Mean number of tables	33.0	7.2
Parties on busiest night of the week		
50-59 parties	25.0 %	2
60-69 parties	37.5 %	3
70+ parties	37.5 %	3
Children’s menu available	75.0 %	6
Restaurant marketing in English only	87.5 %	7
Median weekly sales	$21,500	$8000–$60,000

### Limitations and strengths

A few limitations of the study are acknowledged. Evaluation is limited to implementation, sales and ordering behavior. While the mixed methods are notable, a gap remains in terms of whether introducing healthy children’s menu items in independent restaurants will lead to improvements in dietary consumption. Second, we planned for a 12-week intervention consistent with a recent systematic review [19]. However, budget and time constraints are limiting our study to a 10-week intervention period. Despite these limitations, a strength of this study is its engagement with independent restaurant managers/owners. National efforts focus mainly on chain restaurants, an important but smaller segment of the restaurant market [15]. Ethnic independent restaurants may be the restaurants of choice for certain groups as they seek to retain aspects of their culture of origin. In addition, a previous effort to engage both chain and independent restaurants in modifying their children’s menus was more successful at engaging smaller independent restaurants than chain restaurants [49].

### Conclusions

Spending on FAFH is increasing globally and is not limited to those traditionally exhibiting health disparities. For example, recent evidence from Brazil indicates spending increased by 25 % over a span of four years among individuals of middle and high incomes [56]. Despite evidence that offering healthful items could increase revenue in some types of restaurants (e.g., carry-out) [57], restaurant interventions remain a challenge to implement given managers/owners’ concerns with profit and customer satisfaction [58]. This study lays the groundwork for engaging independent restaurants and testing methods to promote healthy children’s menus. Despite our initial successes, a barrier of engaging parents and children in ordering healthy food while dining out is the belief that going out to eat at restaurants is a treat and/or for enjoyment purposes, and health is not considered on these occasions [42]. This is further compounded by children being allowed to select the restaurant [42] and, as we observed, their menu items. Despite these barriers, McGuffin and colleagues (2014) also noted children and adults were receptive to changes in cooking methods, smaller portion size options, fruits and vegetables as a side, and, more generally, having flexibility when choosing from a healthy menu for children. More research is clearly needed on how to modify consumption of FAFH.

## Ethics approval and consent to participate

All procedures involving human participants were approved by the Institutional Review Board of San Diego State University. If eligibility was met and the restaurant manager/owner agreed, the two parties signed a letter of agreement and confidential disclosure agreement. The manager/owner also signed an informed consent form for the interviews. To obtain consent, dining parties are handed an informational flyer and an informed consent/recruitment script is read by KCRP evaluation staff who then asks if they would be willing to participate and provide permission for their children to participate. If permission is granted, children are asked to provide verbal assent.

## Consent for publication

Not applicable.

## Availability of data and material

The datasets supporting the conclusions of this article are available for request through the IBACH website. Please visit: http://www.ibachsd.org.

## Abbreviations

CA: California
CMA: Children’s Menu Assessment
FAFH: food away from home
FV: fruits and vegetables
ID: Identification
KCRP: Kids’ Choice Restaurant Program
NEMS-R: Nutrition Environment Measurement Survey for Restaurants
SAS: Statistical Analysis Software
SD: standard deviation
SEF: Socio-Ecological Framework
US: United States of America
